# Textural complications of banded iron formation and the potential production of nano-magnetite: a case study from the Central Eastern Desert of Egypt

**DOI:** 10.1038/s41598-023-42058-5

**Published:** 2023-09-13

**Authors:** Mahmoud Abdel-Hakeem, Galal El-Habaak

**Affiliations:** 1 Department of Geology, Faculty of Science, South Valley University, Qena, Egypt; 2 Department of Geology, Faculty of Science, Assiut University, Asyut, Egypt

**Keywords:** Geology, Mineralogy

## Abstract

The current work makes integrated value-added, geological and chemical studies on the texturally intricate banded iron formation “BIF” that is represented here, as a case in point, by the Um Nar BIF located in the Central Eastern Desert of Egypt. Geologically, the Um Nar BIF is composed mainly of oxide-rich facies and silicate-rich facies mostly expressed as bands of variable thickness (90–730 µm). Magnetite, martite, goethite, and quartz are detected as the main components of the oxide-rich facies, while epidote, stilpnomelane, and garnet occupy the other facies type. The studied ore can be classified as a low-grade iron ore containing 51.23 wt.% Fe_2_O_3_ and 39.64 wt.% SiO_2_ along with considerable phosphorous content (1.01 wt.% P_2_O_5_). These elemental concentrations do not match the recommended benchmarks for iron and steelmaking (e.g.75.78–95.8 wt.% Fe_2_O_3_, 5–7 wt.% SiO_2_, and 0.04 wt.% P_2_O_5_). Moreover, the studied BIF has a poor liberation behavior on crushing and grinding due to the complex interlocking of magnetite with quartz and stilpnomelane expressed as a sieve-like texture. This textural complication directed the current work to investigate the potential exploitation of the Um Nar BIF as a precursor of nano-magnetite that is commonly synthesized by ferrous and ferric chlorides. Accordingly, HCl-based agitation leaching followed by co-precipitation was carried out, resulting in ultrafine nano-magnetite (2.47–4.27 nm particle size) expected to serve in water treatment as an effective adsorbent for heavy metals.

## Introduction

Banded iron formations “BIFs” are sedimentary iron ores formed, throughout the Archean and Proterozoic eons (~ 3800–543 Ma), by the deposition of iron minerals-rich bands rhythmically alternative with chert/or jasper bands and considered to be the most important iron source in the world^1^. They have been and still exploited for the production of iron pellets in a number of countries (e.g. Australia, Brazil, and India) after adequate ore dressing processes (e.g. reduction roasting followed by magnetic separation and flotation) due to the lower Fe grades (15–40 wt.%) and the higher SiO_2_ contents (40–60 wt.%)^2–5^. In Egypt, about 53 million tons of the Neoproterozoic BIFs (average 44.37 wt.% Fe_2_O_3_ and 31.45 wt.% SiO_2_) are located at 13 localities (e.g. Um Nar, Wadi Karim, Abu Marwat, Um Ghamis, and Um Anab) through the Central Eastern Desert^6–15^. The metallurgical potential of such considerable tonnages was evaluated by the Egyptian Geological Survey during 6 years (1959–1965) of field, mapping, core drilling, mining investigations, and chemical characterization works. The final decision was that the Eastern Desert BIF cannot be included in the line production of pig iron due to the complex intergrowth between ore and gangue minerals, requiring highly cost separation and concentration processes^16^.

Up to the current date, few studies were conducted on the Egyptian BIF to upgrade the Fe values through different beneficiation processes, including flotation and magnetic separation, which mostly come with unsatisfactory results^17,18^. From this point and as a contribution to literature, the current work tries to attract more attention for the economic potential of the texturally complicated BIFs as a promising source for the production of nano-magnetite. The latter is considered to be an attractive material possessing a wide field of applications including, for example, the magnetic resonance imaging^19^, drug delivery system^20^, cancer therapy^21^, and wastewater treatment^22^. The current approach investigates the extent to which Fe-oxides minerals (e.g. magnetite, martite, and goethite) of BIFs can serve as an alternative nano-magnetite precursor to the commonly used chemicals (e.g. ferrous and ferric chlorides). For this purpose, integrated geological and chemical studies are carried out on the Um Nar BIF, as a case study. The geological work includes textural, mineralogical, and chemical characterizations. On the other hand, the chemical studies comprise a lab-scale agitation leaching of the Um Nar BIF to obtain a high-grade concentrate of nano-magnetite.

## Geology of Um Nar area

**Figure 1:**
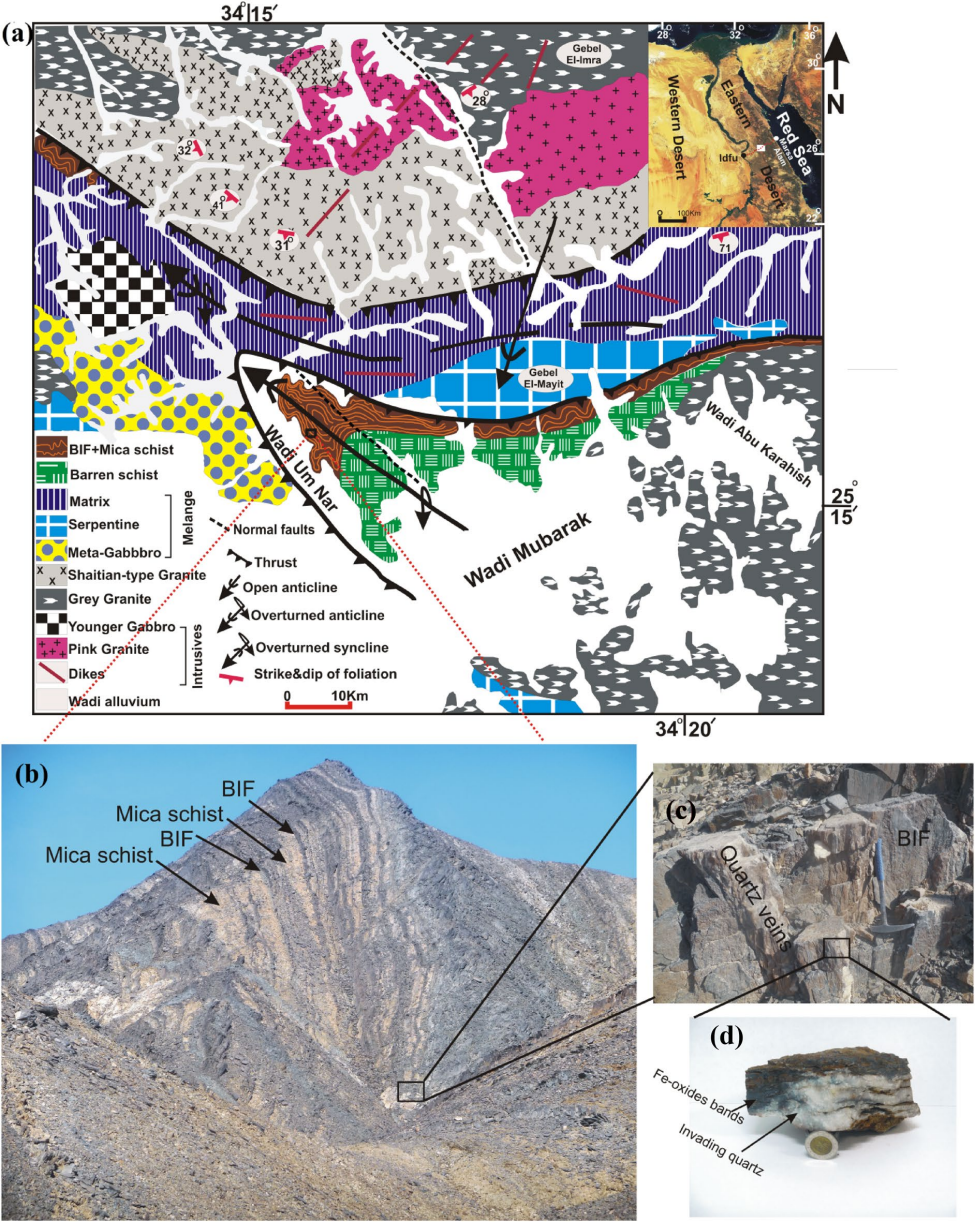
Geological map of the Um Nar area^23^ (**a**), with close-up field views of BIF successions (**b**) that are invaded by quartz veins (white in color) (**c** and **d**).

The Central Eastern Desert “CED” of Egypt is characterized by 13 localities of BIFs, covering about 30,000 km^2^, among which the Um Nar area where 13 million tons of the Neoproterozoic metamorphosed BIF can be encountered along Idfu-Marsa Alam road, between latitudes (25° 14′ 30″  and 25°16′ 24″ N) and longitudes (34° 14′ 50″ and 34° 18′ 56″ E) (Fig. 1a). The Um Nar BIFs are hosted mainly by medium grade, garnet- and epidote-bearing mica schists. Both iron ore and its host rock were regionally metamorphosed during the Pan-African Orogeny, under greenschist-to- amphibolite facies conditions^11^. Morphologically, the studied iron ore is exposed as a thick stratified succession, 300 m thick, of BIFs-rich bands alternating with mica schist, along the southern limb and at the crest of a huge overturned anticline, with NW–SE striking and NE vergence (Fig. 1b). Also, the southern limb is invaded by numerous milky quartz veins and veinlets cross-cut the bedding planes and occasionally replace the bands of Fe minerals (Fig. 1c,d). Moreover, some BIF slivers can be traced along the northern limb. This folding type is considered to be a result of successive deformational events affected the southern part of CED, including arc-arc collision, ophiolite emplacement, collision of East and West Gondwana, and the aforementioned regional metamorphism. In addition to folding, blocks of serpentinite and meta-gabbro along with the ophiolitic mélange of Gebel El Mayit (allochthonous oceanic and continental fragments set in a fine-grained matrix of serpentinite, talc carbonate, and graphite schist) were thrust over the northern limb during the Pan-African Orogeny. The net result of this regional deformation exposes as two overturned folds, comprising the aforementioned BIF-mica schist-bearing anticline and another ophiolitic mélange-bearing syncline, that were thrust over the Shaitian granite. Afterwards, the study area was intruded by varieties of granitic rocks including grey and pink granites^7,8,10,23,24^.

## Methodology

### Characterization studies of BIF

For petrography, fourteen polished sections were prepared and examined under the ore microscope (Olympus-BX5). To obtain one representative sample, the collected BIF samples were homogenized through sequential steps of crushing, milling, and screening up to −1 mm size fraction. The ground sample was split using the riffle divider and about 500 g were taken for the subsequent assays.10 g/sample were hydraulically pressed for performing mineralogical and chemical inspections. X-ray diffractometer (XRD-X'Pert PRO-PAN),with Cu kα (ʎ = 1.540, 40 kV, 25 mA), was adapted to identify the mineralogical composition; the resultant raw data were interpreted using X'Pert High Score Plus software. The chemical composition was determined by X-ray fluorescence hosted at the Egyptian Geological Survey (Sequential WD-XRF). Moreover, the liberation behavior of iron from silica was examined by sieving the ground ore through set of standard sieves (− 1 + 500, − 500 + 250, − 250 + 125, − 125 + 90, − 90 + 45, and − 45 µm). Each size fraction was then weighed and analyzed for iron oxide and silica contents. The effect of comminution and sieving on the liberation of iron oxide was also studied by XRD and grain mounting for each sieve fraction.

### Chemical treatment and synthesis of nano-magnetite

**Figure 2:**
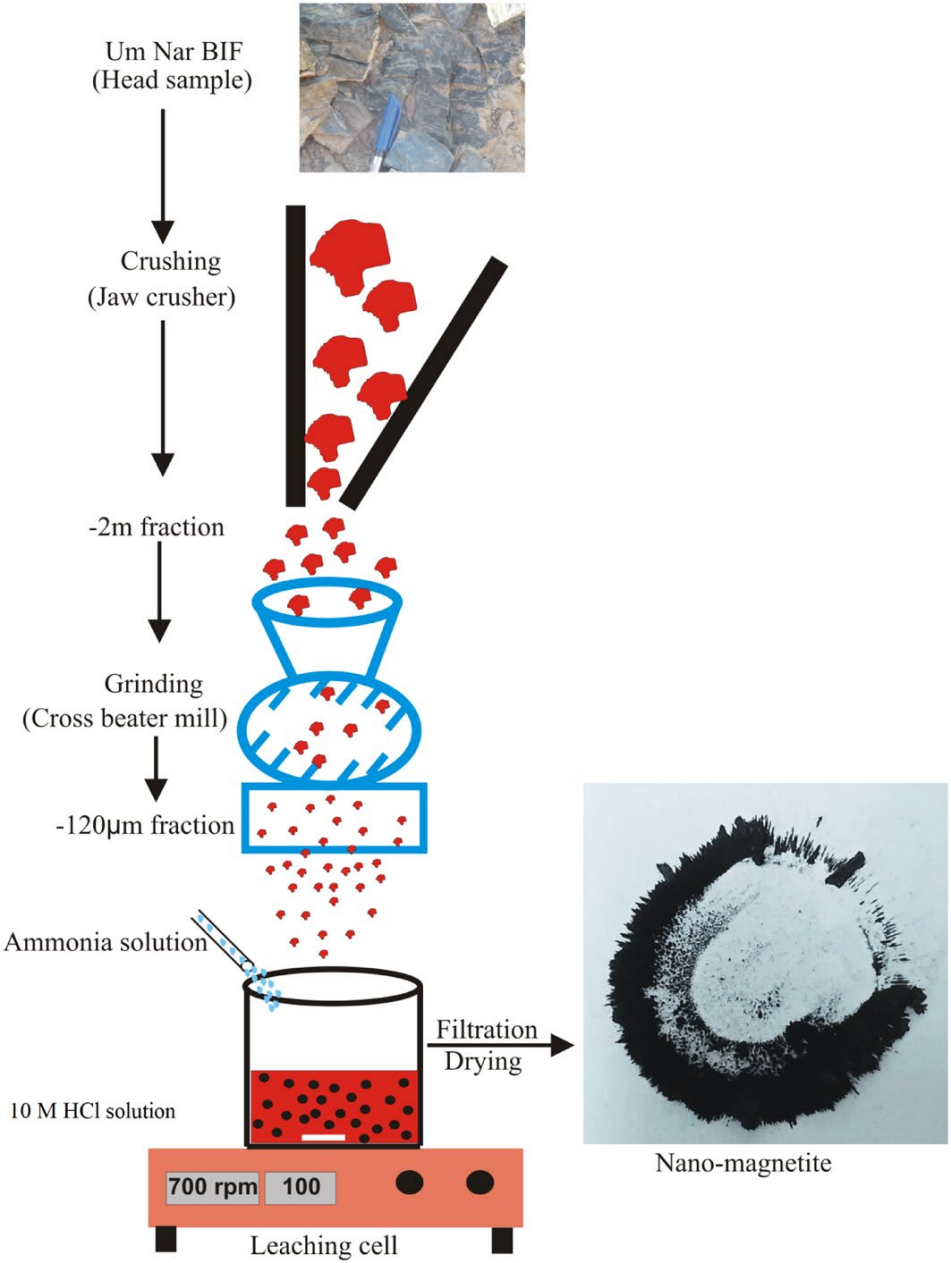
Experimentation for nano-magnetite synthesis.

About 20 kg representative sample of the Um Nar BIF was first crushed and ground using jaw crusher, set at − 2 mm outlet size, and cross beater mill adjusted at − 120 µm output size. The ground ore was then treated using 10 M HCl at 0.2 solid/liquid ratio, 100 °C leaching temperature, and 700 rpm stirring speed for 45 min (Fig. 2). The main aim of this chemical leaching is to obtain a liquid fraction consisting mainly of ferrous and ferric chlorides derived from the dissolution of magnetite, martite, hematite, and goethite. The resultant solution is considered a precursor for the synthesis of non-magnetite. The latter was prepared via co-precipitation method^25,26^ that involved vigorous agitation “700 rpm” for 1 h along with the presence of 25% ammonia solution. The precipitated nano-magnetite was centrifugally obtained, thoroughly washed by de-ionized water, and dried at 70 °C overnight. Afterwards, the chemical composition was studied using the sequential WD-XRF. The crystalline structure was investigated by XRD (X’Pert PRO-PAN) along with Fourier Transform Infrared Spectroscopy “Perkin Elmer-FTIR” through a scanning spectrum of 4000–400 cm^−1^. For particle size, transmission electron microscope “TEM-JEOL” was implemented.

## Results and discussion

### Microscopic characterization

**Figure 3:**
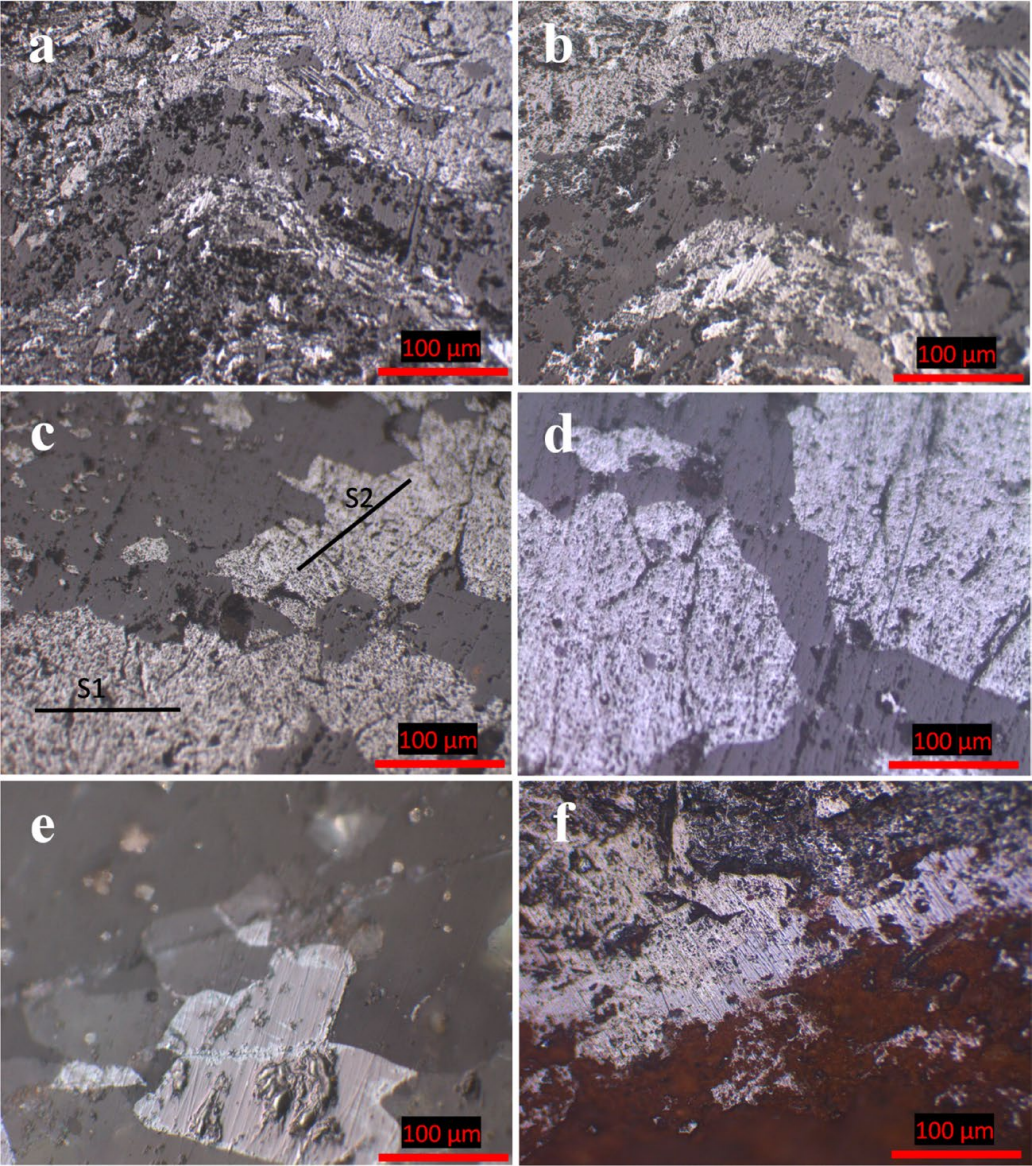
Photomicrographs of Um Nar BIF showing folded stratification (**a** and **b**), S1 along with S2 schistosity of magnetite (**c**), and replacement of magnetite by quartz vienlets (**d**), martite (**e**), and goethite (**f**).

Depending on the microscopic investigations, the Um Nar metamorphosed BIF can be discriminated into oxides-rich facies and silicate-rich facies. The former consists mainly of dark grey jasper bands (90–800 µm thick) rhythmically stratified with light grey bands constituted, in a variable abundance, from magnetite, martite, and goethite. Magnetite (pinkish grey) is considered to be the main unit cell of all observed Fe-bands. In places, this stratification was deformed by the imposed metamorphic conditions, resulting in micro- and macroscopic folds (Fig. 3a,b). Besides folding, the deformational structures are represented by two types of schistosity^23^. The former one is called stratification parallel schistosity “S_1_” that can be traced through idiomorphic, sub-idiomorphic, and xenomorphic magnetite crystals (60–220 µm in diameter) arranged with each other in the form of stratiform, connected or sporadic laminae. The latter type “S_2_” is associated with the folding process as axial plane schistosity and inclined in relative to S_1_ (Fig. 3c). With increasing the metamorphic grade, the aforementioned magnetite crystals were recrystallized into coarse-grained porphyroblasts (300–650 µm in diameter) that frequently crowd with each other to form magnetite bands (190–730 µm thick) (Fig. 3c). The latter is occasionally invaded by quartz veinlets (Fig. 3d). In all cases, magnetite shows noticeable signs for variable degree of martitization. For instance, both granular and banded magnetite forms, in places, are oxidized along peripheries and octahedral planes into light grey martite (Fig. 3e). Like martite, goethite, bluish grey, is regarded as an alteration product of magnetite. It appears as irregular patches through magnetite bands (Fig. 3f).

**Figure 4:**
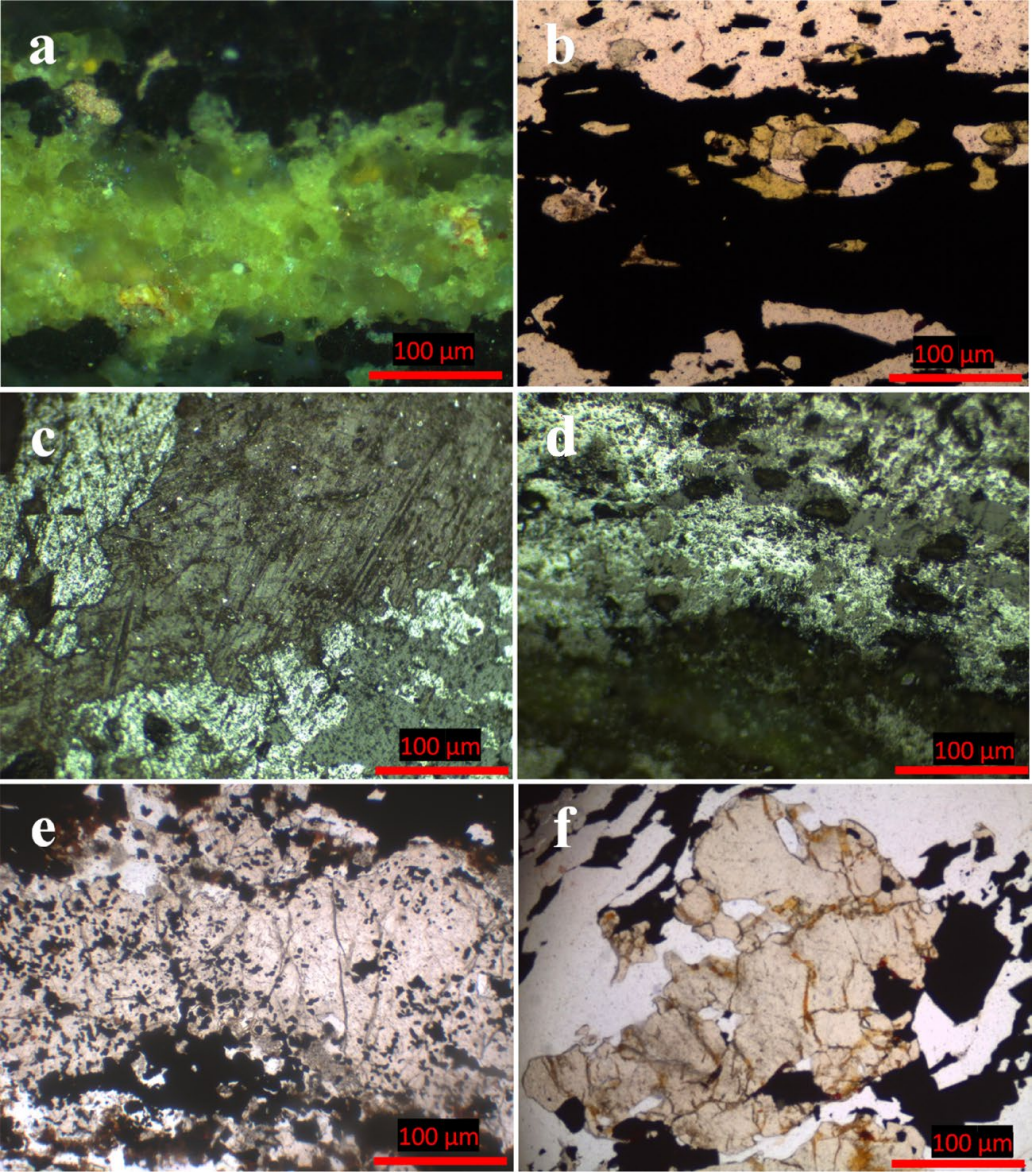
Photomicrographs showing the replacement of magnetite bands by epidote (**a** and **b**) and stilpnomelane (brownish grey) (**c** and **d**) as well as garnet as bands (**e**) and rotated grains along S2 (**f**).

Regarding silicate-rich facies, epidote, garnet, and stilpnomelane are detected as the principle mineral components, with subordinate amounts of martitized magnetite. Epidote exhibits a distinguishable green internal reflection and appears like stratiform bands varying in thickness between 150 and 330 µm (Fig. 4a). It also occurs as aggregates commonly replacing magnetite crystals (Fig. 4b). Stilpnomelane, brownish grey in color, is considered to be the only iron silicate mineral that can be clearly noticed in the studied iron ore. It is observed as fracture infill through magnetite bands (Fig. 4c) as well as thin laminae after magnetite (Fig. 4d). Garnet appears as coarse-grained porphyroblasts, containing inclusions of quartz and magnetite, sometimes coalesce with each other to form lensoidal bands (Fig. 4e). In places, garnet porphyroblasts are arranged parallel to S2 direction, indicating some rotation degree (Fig. 4f).

**Figure 5:**
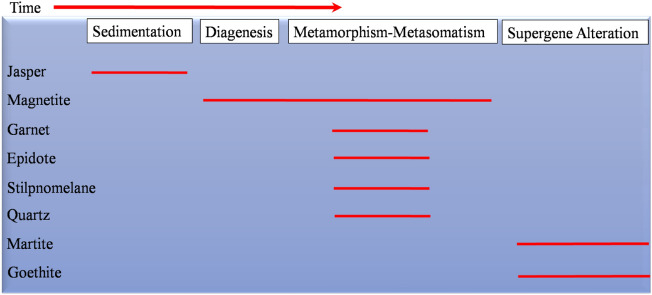
Mineral paragenesis of the Um Nar BIF.

Paragenetically, the above mentioned minerals have been formed during four main stages: (a) sedimentation, (b) diagenesis, (c) metamorphism, and (d) supergene alteration (Fig. 5). During deposition stage, bands of jasper “the first generation of silica” and Fe-oxyhydroxides settled down, producing rhythmic stratification. Subsequently, the diagenetic conditions developed the primitive bands of Fe-oxyhydroxides into the first generation of magnetite. After a specific period of time, the Um Nar area was regionally metamorphosed under greenschist-amphibolite facies conditions. This is clearly manifested by the occurrence of garnet and epidote. Moreover, the imposed metamorphic conditions were associated with hydrothermal solutions, resulting in metasomatic magnetite porphyroblasts “the second generation”, stilpnomelane, and quartz vienlets “the second generation of silica”. Eventually, the metamorphosed BIF experienced some degree of the supergene alteration, causing the formation of martite and goethite at the expense of magnetite.

**Figure 6:**
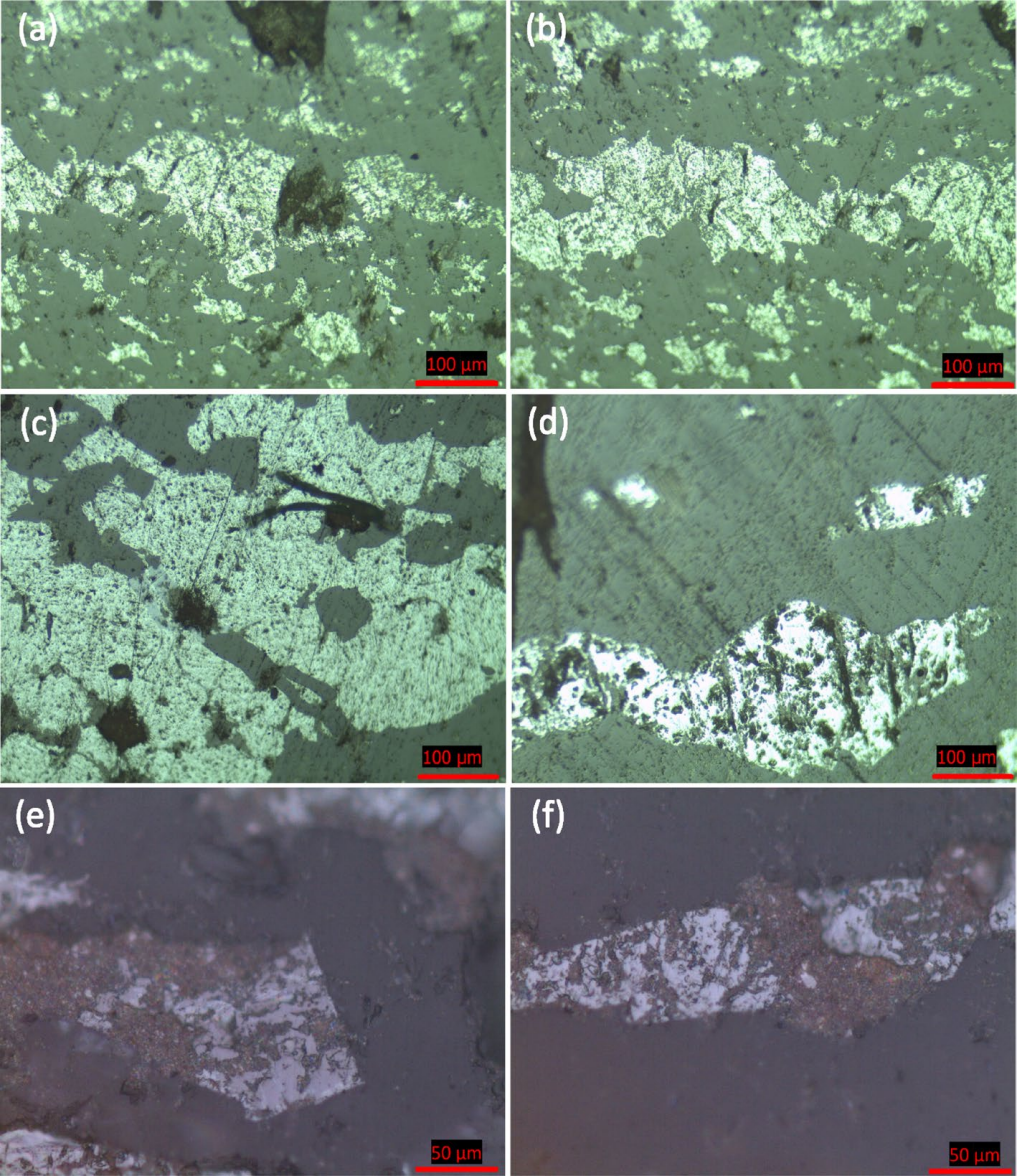
Photomicrographs showing the complicated texture of Um Nar BIF represented by sieving and corrosion of the martitized magnetite (light grey) by quartz (dark grey) (**a**–**c**) and stilpnomelane (**d**–**f**).

From an applied perspective, the microscopic investigations of the low-grade ore deposits, in particular the textural relationships between valuable and gangue minerals, can make a crucial decision about the amenability of these ores to undergo mineral beneficiation at a profit. As aforementioned, the studied iron ore generally exhibits rhythmically stratified bands. Consequently, crushing of these bands followed by suitable concentration process (e.g. magnetic concentration, flotation, and gravity separation) seems, at first glance, an effective procedure for Fe-minerals separation. However, the detailed microscopic inspection has showed that the majority of martitized magnetite bands are corroded and sieved by quartz (Fig. 6a–c) and stilpnomelane (Fig. 6d–f) whose complex interlocking with magnetite makes the separation process more difficult. Magnetite corrosion by quartz is well documented through the previously mentioned field observations (Fig. 1c,d); it can be explained by the fact that magnetite is less stable under acidic conditions, represented here by silica-rich hydrothermal solutions, which promoted Si^4+^ substitution for Fe^3+^ in both the tetrahedral and octahedral sites^27,28^. Moreover, the released ferric iron is thought to be implicated in the formation of stilpnomelane aggregates that fill many micropores of the corroded magnetite. Due to its content of ferrous iron, stilpnomelane is thought to be separated along with Fe-oxide minerals once adequate magnetic field is applied.

### Mineralogical characterization of BIF

**Figure 7:**
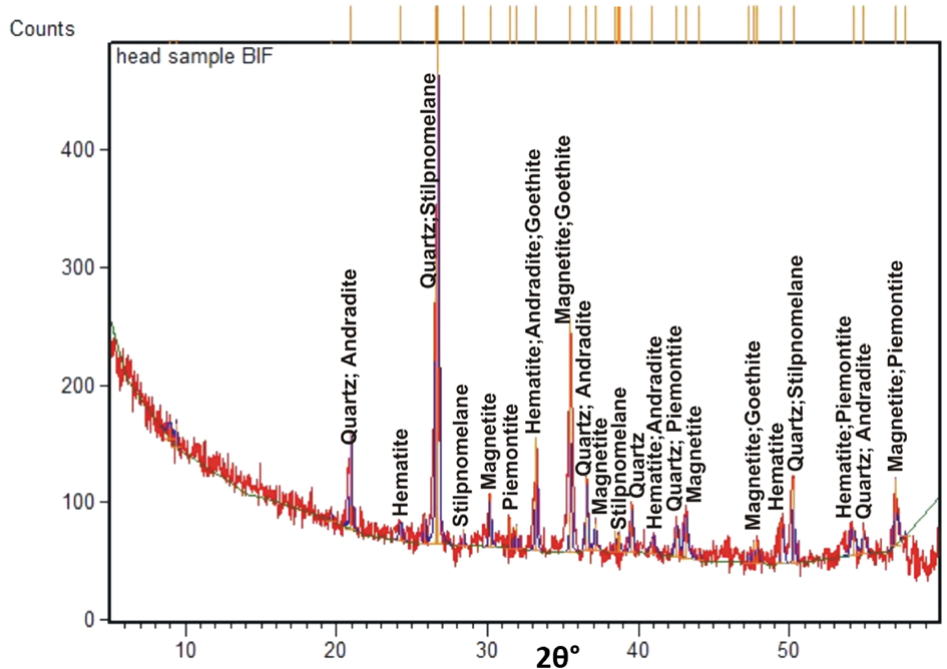
XRD pattern of the head ore sample.

For head sample, interpretation of XRD pattern (Fig. 7) supports the mineralogical data previously provided by the optical microscope, in terms of the occurrence of magnetite, martite (hematite), goethite, quartz, stilpnomelane, epidote (piemontite), and garnet (andradite). Stilpnomelane occupies the highest reflection peak rather than magnetite, indicating the intensive corrosion and transformation of magnetite into iron silicate. Also, martitization process is well manifested by the presence of hematite peaks.

### Chemical characterization of BIF

The distribution of major oxides was studied for the Um Nar BIF before and after the chemical treatment, as listed in Table 1. The major occurrence of Fe-oxides (magnetite, martite/hematite, and goethite) along with stilpnomelane, quartz, epidote, and garnet in the Um Nar BIF results in about 95.04 wt.% of the chemical composition is dominated by Fe_2_O_3_ (51.23 wt.%), SiO_2_ (39.64 wt.%), and CaO (4.17 wt.%), with a lesser content (≈ 4.03 wt.%) of the other oxides including TiO_2_ (0.16 wt.%), MgO (0.42 wt.%), MnO (0.08 wt.%), Al_2_O_3_ (2.52 wt.%), P_2_O_5_ (1.01 wt.%), and SO_3_ (0.48 wt.%)).Such elemental distribution is greatly similar to the average composition of the Algoma and Lake Superior banded iron formations, the two main BIF-types all over the world^1,29^.

**Table 1:** Distribution of major oxides (wt.%) of the Um Nar BIF, before chemical treatment, compared to Algoma- and Lake Superior-types along with the recommended specifications for the iron and steelmaking^33–35^.

Oxides (wt.%)	Um Nar BIF (Egypt)	Algoma-type BIF (Oxide-facies)	Lake superior-type BIF (Oxide-facies)	Specifications for steelmaking
SiO_2_	39.64	51.58	49.13	5–7
TiO_2_	0.16	0.09	0.02	–
Al_2_O_3_	2.52	3.06	1.45	3–4
Fe_2_O_3_	51.23	42.62	46.33	75.78–95.8
MnO	0.08	0.14	0.76	1.89
MgO	0.42	1.56	1.29	–
CaO	4.17	1.54	1.64	–
Na_2_O	ND	0.32	0.12	–
K_2_O	0.03	0.59	0.15	–
P_2_O_5_	1.01	0.21	0.06	0.04
SO_3_	0.48	0.30	0.02	0.6

Regarding ore grade, the studied BIF can be classified as a low-grade iron ore whose total iron content varies between 20 and 47 wt.% along with higher grades of alumina and silica^30^. By comparing with the ideal benchmarks of iron ores recommended for the iron and steelmaking (Table 1), the Um Nar BIF cannot be directly exploited as a feed material for the iron blast furnace, but instead adequate and appropriate beneficiation processes are first required. For more instance, the Um Nar BIF contains lower concentrations of iron (35.82 wt.%) and higher levels of silica (39.64 wt.%). The latter adversely affects the performance of blast furnace by producing a viscous slag that requires higher flux addition and also makes the removal of steel scale difficult during pickling process^31,32^. Alumina and manganese are other deleterious impurities that contribute to increase the viscosity of furnace slag and the consumption of coke^33^; however, allowable contents of such elements are detected in the studied iron ore. On the other hand, phosphorous is measured at a higher content (1.01 wt.%), making the Um Nar BIF as a high-phosphorous iron ore (> 0.1 wt.% P_2_O_5_), and hence the formation of iron phosphides during the downstream reduction processes and the subsequent production of brittle steel are expected^34,35^.

### Liberation behavior

**Figure 8:**
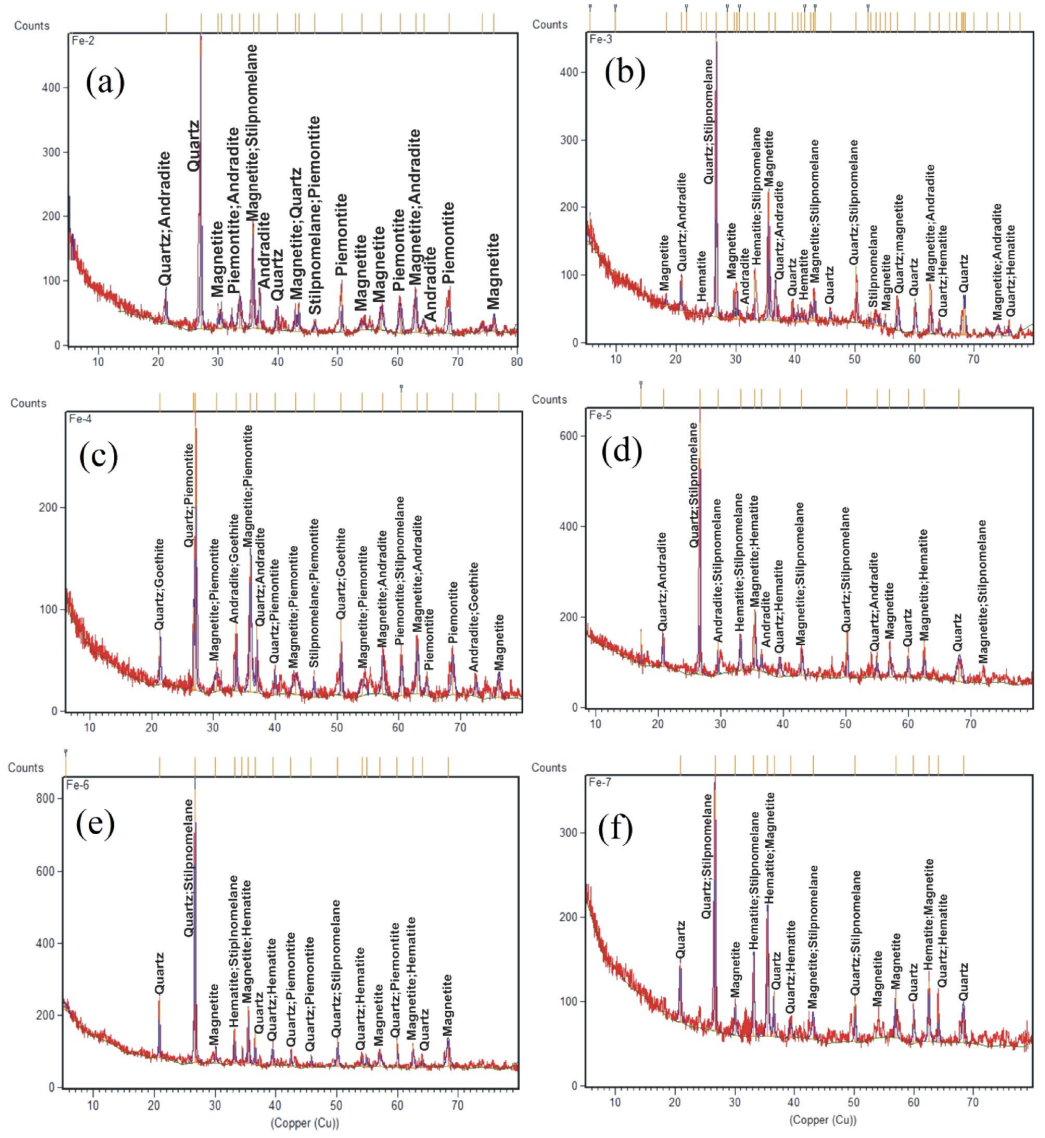
XRD patterns of the different size fractions (**a**-500 µm, **b**-250 µm, **c**-125 µm, **d**-90 µm, **e**-45 µm, and f- < 45 µm).

**Figure 9:**
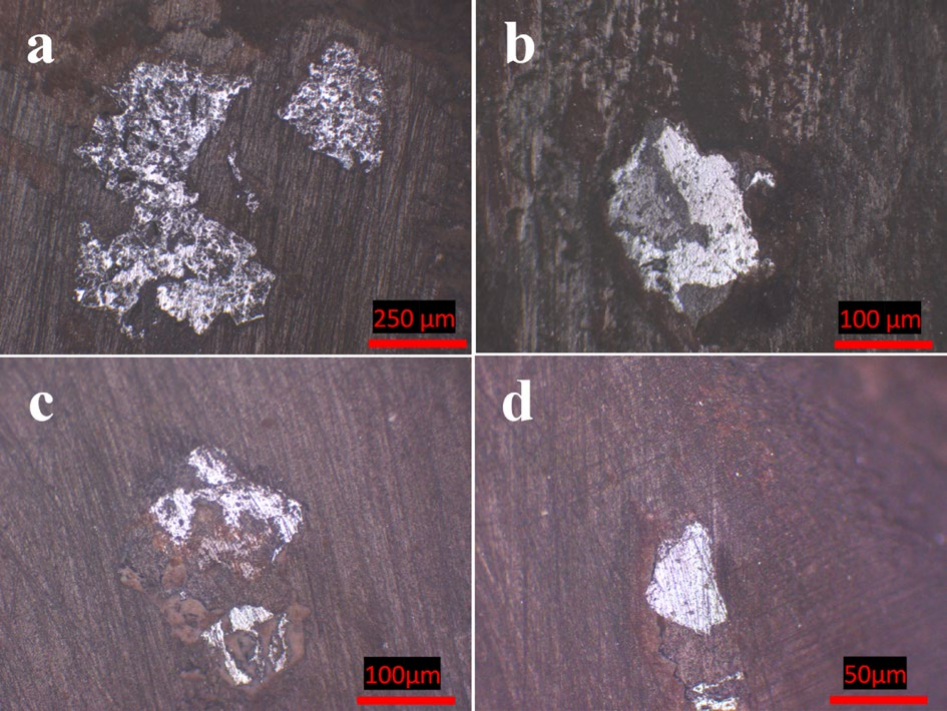
Photomicrographs of some grain mounts (**a**-500 µm, **b**-250 µm, **c**-125 µm, **d**-90 µm) showing the complex libration between magnetite and silica.

**Figure 10:**
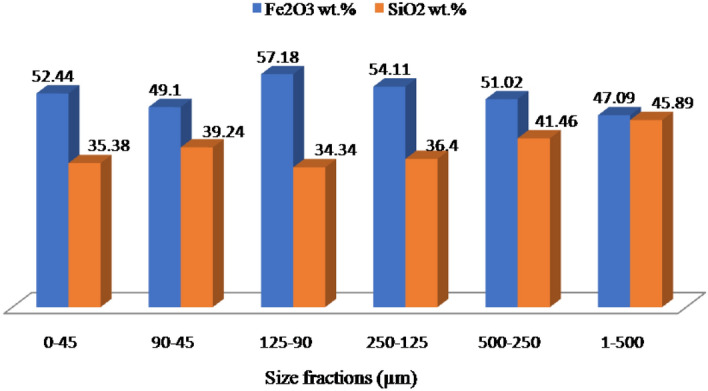
Distribution of Fe_2_O_3_ and SiO_2_ contents through the different size fractions.

From a personal point of view, the term liberation can be defined as the breakage of interlocking, after adequate size reduction, between valuable and gangue minerals. This procedure is considered to be a crucial step on which the decision can be made for the exploitation of mineral resources. The liberation behavior of the Um Nar BIF was studied using successive steps, including grain size analysis, XRD, grain mounts, and chemical assays of Fe “Fe_2_O_3_” and silica contents for each size fraction. For grain size analysis (Table 2), about 52.6 wt.% of the ground sample is rested on 500 µm (24.21 wt.%) and 45 µm mesh sizes (28.39 wt.%). The remnant fraction (47.4 wt.%) is distributed through 250 µm (14.24 wt.%), 125 µm (16.47 wt.%), 90 µm (10.3 wt.%), and − 45 µm size fractions (6.39 wt.%). All of these mesh sizes underwent XRD assays that revealed the continuous appearance of quartz and alumino-silicates (stilpnomelane, andradite, and epidote “piemontite”) along with Fe-oxides minerals (Fig. 8). These assays were considered as the first signs for a difficult liberation behavior. In addition to XRD, the grain mounts (Fig. 9) reveal the complex interlocking between magnetite and silica, indicating the weak effect of size reduction on the liberation degree. The latter was also monitored by plotting each size fraction against the contents of Fe_2_O_3_ and SiO_2_ (Fig. 10). It was found that the finer size fractions “ < 250 µm” contain most of the higher Fe grades (e.g. 57.18 wt.% at + 90 µm particle size) and the lower silica contents (e.g. 34.34 wt.% at + 90 µm) compared to the coarser fractions (e.g. + 500 µm fraction assaying 47.09 wt.% Fe_2_O_3_ and 45.89 wt.% SiO_2_). This is likely pertained to the difference in hardness between silica and Fe-oxide minerals. However, the levels of silica are largely comparable through all finer fractions (e.g. ranging between 39 and 34 wt.%), indicating a poor liberation behavior of the studied ore and supporting the aforementioned microscopic investigations.

**Table 2:** Grain size analysis of the ground Um Nar BIF along with the distribution “wt.%” of Fe_2_O_3_, SiO_2_, Al_2_O_3_, and CaO through the different size fractions.

Fractions (µm)	Wt.%	Fe_2_O_3_	SiO_2_
− 1, + 500	24.21	47.09	45.89
− 500, + 250	14.24	51.02	41.46
− 250, + 125	16.47	54.11	36.40
− 125, + 90	10.3	57.18	34.34
− 90, + 45	28.39	49.10	39.24
− 45 (Pan)	6.39	52.44	35.38

### Characterization of nano-magnetite

#### Chemical characterization

First of all, the synthesis of nano-magnetite was associated with noticeable color transformations starting with a nascent reddish brown precipitate at ~ 4 pH and terminating once a black colored precipitate become dominant at 10.5 pH. This phenomenon can be explained according to the hypothesis of Mascolo et al.^36^. Through this context, the nano-magnetite is initiated as a precipitate of ferric hydroxide “Fe (OH)_3_” that gradually dissociates, with increasing alkalinity, into FeOOH “solid”. The latter reacts with the precipitated ferrous hydroxide “Fe (OH)_2_” to form magnetite. In all cases, the obtained nano-magnetite fraction accounts for 51.79 wt.% of the starting ore sample along with 48.21 wt.% for silica-dominated fraction. The former is recovered at 98.45% and composed mainly of 97.37 wt.% Fe_2_O_3_, with impurities content, including 0.51 wt.% SiO_2_, 0.44 wt.% Al_2_O_3_, 0.17 wt.% Mn, 0.08 wt.% P_2_O_5_, and 0.24 wt.% SO_3_ (Table 3). Moreover, the synthesized nano-magnetite can be chemically correlated with the commonly used adsorbents of Fe-oxides consisting of 98.63wt.% Fe_2_O_3_, 0.44 wt.% SiO_2_, 0.45 wt.% Al_2_O_3_, and 0.12 wt.% CaO^37^. On the other hand, the rest of Fe recovery “1.55%” is included in the insoluble silica fraction at 1.65 wt.% Fe_2_O_3_ along with the main constituents of SiO_2_ “89.37 wt.%”, Al_2_O_3_ “3.81 wt.%”, and CaO “3.74 wt.%”.

**Table 3:** Distribution of major oxides “wt.%” for nano-magnetite concentrate and silica fraction comparing to the commonly used Fe-oxides adsorbents^37^.

Oxides	SiO_2_	TiO_2_	Al_2_O_3_	Fe_2_O_3_	MnO	MgO	CaO	Na_2_O	K_2_O	P_2_O_5_	SO_3_
Mg. Con	0.51	0.13	0.44	97.37	0.17	0.25	0.56	ND	0.03	0.08	0.27
Fe.Ads	0.44	–	0.45	98.63	–	–	0.12	–	–	–	–
Si. Frac	89.37	0.05	3.81	1.64	0.001	0.16	3.74	ND	0.15	0.33	0.87

Mg. Con., nano-magnetite concentrate; Fe. Ads., commonly used Fe-oxides adsorbents; Si. Frac., silica fraction.

#### Structural characterization

**Figure 11:**
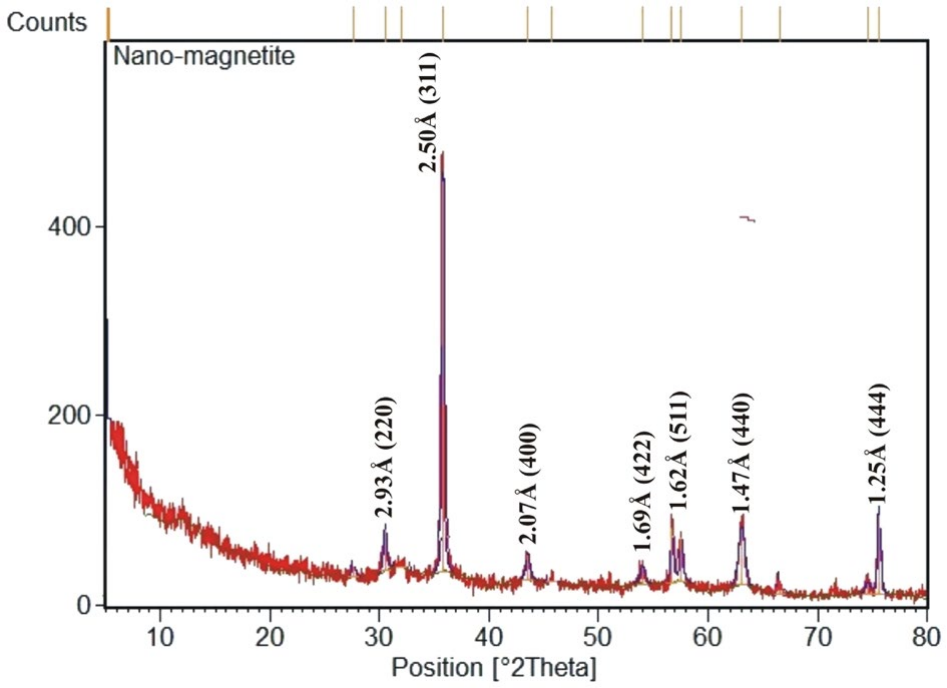
XRD pattern of the resultant nano-magnetite showing the characteristic d-space values and crystalline planes.

**Figure 12:**
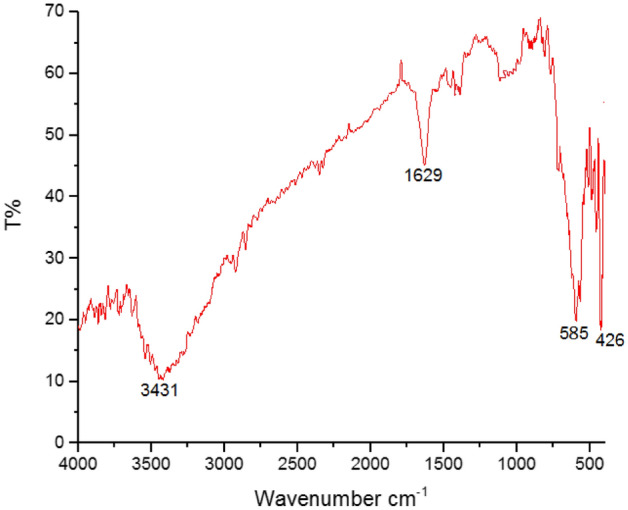
FTIR spectrum of the precipitated nano-magnetite showing the two characteristic absorption bands at 585 cm^−1^ and 426 cm^−1^.

The nano-magnetite concentrate was structurally studied using XRD and FTIR. The interpretation of XRD pattern (Fig. 11) revealed that the nano-magnetite is the dominant constituent characterized by seven diffraction peaks appear at d-space values of 2.93, 2.50, 2.07, 1.69, 1.62, 1.47, and 1.25 Å. For crystallinity, it can be said that a well-crystalline nano-magnetite was yielded with remarkable sharp peaks whose crystalline planes (220), (311), (400), (422), (511), (440), and (444) indicate a cubic spinel structure^25^. Besides XRD, FTIR was used to focus more light on the crystalline structure of nano-magnetite. As shown in Fig. 12, two strong absorption bands at 585 and 426 cm^−1^ are characteristic of this spectrum and can be assigned to the stretching mode of Fe–O bonds at the tetrahedral and octahedral sites, respectively^26,38,39^. Also, the vibration band at 1629 cm^−1^ was ascribed in literature to Fe–O bonds^40^. On the other hand, the synthesized nano-magnetite adsorbs H_2_O molecules as moisture content that expressed by the stretching vibration of OH groups at 3431 cm^−1^.

#### Particle size distribution

**Figure 13:**
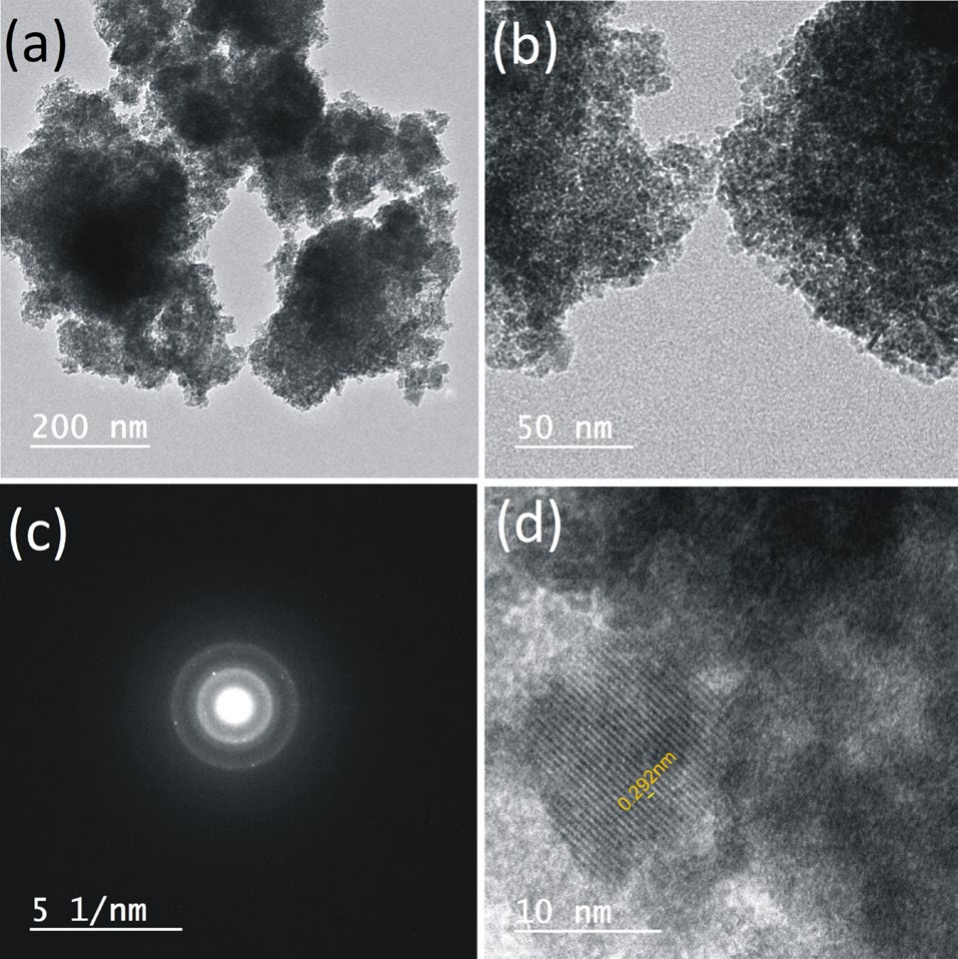
TEM images of the nano-magnetite crystals at different magnifications (**a** and **b**) along with SAED pattern (**c**) and lattice fringes of a single crystal (**d**).

For particle size, TEM inspection at different magnifications (Fig. 13a,b) reveals the granular dispersion of nano-magnetite at particle size varying between 2.47 nm and 4.27 nm, along with SAED pattern of nano-magnetite single crystal (Fig. 13c) and the characteristic 0.292 nm lattice fringes (Fig. 13d) of the cubic spinel structure of magnetite^41,42^. This ultrafine particle size enables the synthesized nano-magnetite to be well dispersed in water and hence can be used as a promising adsorbent for many heavy metal ions (e.g. Pb^2+^, Cu^2+^, Ni^2+^, and As^3+^)^43^.

## Conclusion

The Um Nar BIF, like its counterparts of the metamorphosed banded iron ores, has a complex interlocking between iron minerals and gangues due to the imposed metamorphic events (e.g. recrystallization processes and hydrothermal replacement). Besides the textural complications, the studied ore contains lower grades of iron “51.23 wt.% Fe_2_O_3_” and higher levels of silica “39.64 wt.% SiO_2_”. Consequently, the Um Nar metamorphosed BIFs are unfavorable for the metallurgical purposes. However, the current study has proven that there is another chance for these ores to be valorized in other industrial applications. The occurrence of ferrous and ferric iron minerals, including magnetite, martite, and goethite, has shed more light on the possible exploitation of the texturally complicated BIF as a precursor for the production of nano-magnetite through agitation leaching followed by co-precipitation method. Actually, it was only 45 min agitation time, in the presence of 10 M HCl, sufficient for recovery about 98.45% of the Um Nar BIF as nano-magnetite. The latter was obtained at high purity levels “97.37 wt.% Fe_2_O_3_” that can be comparable to the commercial iron oxides. Furthermore, it is distinguished by ultrafine particle size varying between 2.47 and 4.27 nm. The obtained nano-magnetite can be used as an effective adsorbent for water treatment applications.

## Data Availability

The datasets used and/or analysed during the current study available from the corresponding author on reasonable request.
