# Author Correction: Textural complications of banded iron formation and the potential production of nano-magnetite: a case study from the Central Eastern Desert of Egypt

**DOI:** 10.1038/s41598-024-66735-1

**Published:** 2024-07-16

**Authors:** Mahmoud Abdel‑Hakeem, Galal El‑Habaak

**Affiliations:** 1 Department of Geology, Faculty of Science, South Valley University, Qena, Egypt; 2 Department of Geology, Faculty of Science, Assiut University, Asyut, Egypt

Correction to: *Scientific Reports* 10.1038/s41598-023-42058-5, published online 13 September 2023

The original version of this Article contained errors. The represented data for the surface area and pore size of the resultant nano-magnetite were incorrect.

In the Abstract,

“Accordingly, HCl-based agitation leaching followed by co-precipitation was carried out, resulting in ultrafine mesoporous nano-magnetite (2.47–4.27 nm particle size, 120 m^2^g^−1^ surface area, 0.55 cm^3^g^−1^ pore volume, and 4.88 nm pore diameter) expected to serve in water treatment as an effective adsorbent for heavy metals.”

now reads:

“Accordingly, HCl-based agitation leaching followed by co-precipitation was carried out, resulting in ultrafine nano-magnetite (2.47–4.27 nm particle size) expected to serve in water treatment as an effective adsorbent for heavy metals.”

In the Methodology, under the subheading ‘Chemical treatment and synthesis of nano-magnetite,

“For particle size, transmission electron microscope “TEM-JEOL” was implemented. Also, the total surface area and the distribution of pore size were studied by N_2_-adsorption BET method that performed using Micromeritics ASAP 2020 at the National Research Center, Egypt.”

now reads:

“For particle size, transmission electron microscope “TEM-JEOL” was implemented.”

In the Results and discussion, under the subheading ‘Characterization of nano-magnetite’, “Surface area and pore size distribution” subsection was removed. Subsequently, Figures 14 and 15 and References 44 and 45 were removed.

Finally, in the Conclusion,

“Furthermore, it is distinguished by ultrafine particle size varying between 2.47 and 4.27 nm, pore volume of 0.55 cm^3^ g^−1^, uniform pore diameter measured at 4.88 nm, and 120 m^2^ g^−1^ total surface area. Accordingly, the obtained nano-magnetite can be classified as mesoporous nano-particles that can be used as an effective adsorbent for water treatment applications.”

now reads:

"Furthermore, it is distinguished by ultrafine particle size varying between 2.47 and 4.27 nm. The obtained nano-magnetite can be used as an effective adsorbent for water treatment applications."

The original Article has been corrected.

